# Identification and first characterization of DinJ-YafQ toxin-antitoxin systems in *Lactobacillus* species of biotechnological interest

**DOI:** 10.1038/s41598-019-44094-6

**Published:** 2019-05-21

**Authors:** Alberto Ferrari, Stefano Maggi, Barbara Montanini, Alessia Levante, Camilla Lazzi, Yoshihiro Yamaguchi, Claudio Rivetti, Claudia Folli

**Affiliations:** 1 Department of Food and Drug, University of Parma, 43124 Parma, Italy; 2 Department of Chemistry, Life Sciences and Environmental Sustainability, University of Parma, 43124 Parma, Italy; 3 The OCU Advanced Research Institute for Natural Science and Technology (OCARINA), Osaka City University, Sumiyoshi-ku, 558-8585 Osaka, Japan

**Keywords:** Bioinformatics, Bacterial toxins

## Abstract

DinJ-YafQ is a type II TA system comprising the ribosome-dependent RNase YafQ toxin and the DinJ antitoxin protein. Although the module has been extensively characterized in *Escherichia coli*, little information is available for homologous systems in lactic acid bacteria. In this study, we employed bioinformatics tools to identify DinJ-YafQ systems in *Lactobacillus casei*, *Lactobacillus paracasei* and *Lactobacillus rhamnosus* species, commonly used in biotechnological processes. Among a total of nineteen systems found, two TA modules from *Lactobacillus paracasei* and two modules from *Lactobacillus rhamnosus* wild strains were isolated and their activity was verified by growth assays in *Escherichia coli* either in liquid and solid media. The RNase activity of the YafQ toxins was verified *in vivo* by probing mRNA dynamics and metabolism with single-cell Thioflavin T fluorescence. Our findings demonstrate that, albeit DinJ-YafQ TA systems are widely distributed in lactic acid bacteria, only few are fully functional, while others have lost toxicity even though they maintain high sequence identity with wild type YafQ and a likely functional antitoxin protein.

## Introduction

Toxin-antitoxin (TA) systems are widely prevalent in plasmids and chromosomes of bacteria and archaea, playing an important role in regulating cell growth and death under various stress conditions. All these systems encompass a stable toxin, a peptide or a protein capable of inducing cell death or dormancy, and an unstable antitoxin, a non-coding RNA or a protein, that neutralizes the toxin activity. TA systems are classified in six different groups, designated type I-VI on the basis of the antitoxin nature and mechanism of action^1^. In type II TA systems, toxin and antitoxin are both proteins that form a stable inactive complex under normal growth conditions. However, under stress conditions the unstable antitoxin is degraded by cellular proteases leaving the toxin molecules able to interfere with different cellular processes, such as DNA replication, RNA transcription, protein and peptidoglycan synthesis. Interference with the cell molecular machinery causes growth arrest that lasts as long as the stress persists. TA system activity is indeed part of an adaptive response mechanism of the bacteria which allows the population to survive under adverse conditions. This cellular dormant state is particularly studied in bacteria exposed to antibiotics and it is known as persistence^1–3^. However, little knowledge is available about the presence of TA systems in lactic acid bacteria (LAB). To date, a few TA systems located on the chromosome or plasmids of LAB have been identified by bioinformatics analysis and characterized for their activity^4–6^. LAB are widely used in industrial applications and are an important component of the human microbiota. Fermented food is an hostile environment for bacteria because of unfavorable conditions (nutrient scarcity, low pH, high salt concentration, low oxygen concentration), thus TA systems may represent mechanisms that promote bacterial adaptation by controlling survival and growth.

Among type II TA systems, the best characterized toxins are ribonucleases which are classified as ribosome-dependent mRNA interferases or ribosome-independent mRNA interferases^7^. The DinJ-YafQ system, whose genes constitute an operon with the antitoxin gene (*dinJ*) located upstream of the toxin gene (*yafQ*), has been extensively studied in *Escherichia coli*. The expression of this TA operon is regulated by the antitoxin DinJ and by DinJ-YafQ complex that repress transcription upon binding to a palindromic sequence partially overlapped with the operon promoter elements^8,9^. In addition, this regulatory region contains a LexA-binding consensus sequence indicating that the expression may also be activated under DNA damage SOS response^10^. The YafQ toxin interacts with ribosome probably by recognizing the 16S rRNA and cleaves a specific mRNA sequence in the A site^11^. Determination of *E*. *coli* YafQ and (DinJ)_2_-(YafQ)_2_ heterotetrameric complex crystal structures, provided insights into the catalytic site of the toxin and revealed structural details of the toxin-antitoxin interaction^9,12^. The antitoxin DinJ folds into three domains: a N-terminal domain that forms the homodimer and is involved in promoter recognition, a middle linker domain and a C-terminal domain that interacts with the YafQ toxin. The (DinJ)_2_-(YafQ)_2_ complex forms a triangle-shaped structure with the DinJ dimer at the apex interacting with two YafQ molecules. YafQ shows a globular structure consisting of a β-sheet (β1–β4) surrounded by 3 α-helices; structural studies associated with mutagenesis experiments have identified in a cleft between the β-sheet (β2–β4) and helix α3 the catalytic site of the toxin. In *E*. *coli*, the comparison between YafQ and the complexes of RelE and YoeB bound to 70S ribosome^11,13,14^, suggests a common mechanism of action of these endoribonucleases and leads to the identification of the YafQ amino acids likely involved in the catalysis (H50, H63, H87, D61, D67 and F91). Furthermore, mutagenesis experiments have shown that the substitutions H50A, H63A, H87A, D67A and F91A abolish toxin activity *in vivo*, whereas the substitution D61A reduces toxin activity by ~50%^15^.

In this work, we used bioinformatics tools to analyse the distribution in lactic acid bacteria of type II TA systems comprising a ribosome-dependent mRNA interferase toxin. Among the TA systems found, DinJ-YafQ and YefM-YoeB are by far the most frequent but, within the *Lactobacillus* species *casei*, *paracasei* and *rhamnosus*, largely used in biotechnology, DinJ-YafQ is the most represented. Among the identified TA systems, we have verified the activity in *E*. *coli* of two DinJ-YafQ systems isolated from wild strains of *L*. *paracasei* and *L*. *rhamnosus* by using growth assays and single cell fluorescence microscopy.

## Results

### Distribution of the DinJ-YafQ and YefM-YoeB toxin-antitoxin systems in lactic acid bacteria

**Figure 1:**
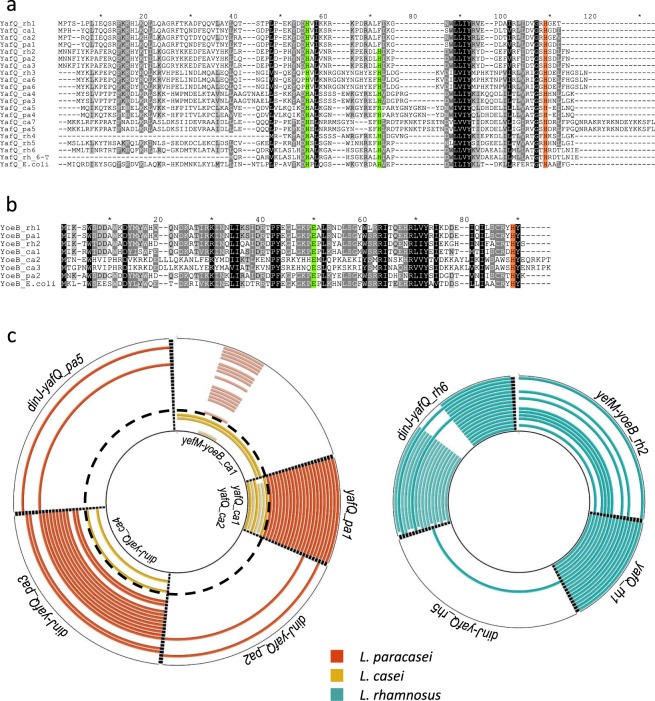
Identification of *dinJ-yafQ* and *yefM-yoeB* TA systems in *L*. *casei*, *L*. *paracasei* and *L*. *rhamnosus*. (**a**) Sequence alignment of YafQ toxins retrieved from PSI-BLAST with *E*. *coli* YafQ (UniProt Q47149); YafQ_rh6-T represents the truncated form of YafQ_rh6. (**b**) Sequence alignment of YoeB toxins retrieved from PSI-BLAST with *E*. *coli* YoeB (UniProt P69348). Catalytic residues are highlighted in color: predicted general base residues in green; predicted general acid in orange. (**c**) BRIG plot showing the distribution of *dinJ-yafQ* and *yefM-yoeB* TA systems in chromosomal DNA of *L*. *casei* (yellow), *L*. *paracasei* (orange) and *L*. *rhamnosus* (green).

The main TA systems comprising a ribosome-dependent RNA interferase toxin are DinJ-YafQ, YafNO, HigBA, RelBE and YefM-YoeB^7^. To assess the distribution of these TA systems in *Lactobacillus* genus, a Blastp search was performed by using toxin sequences from *E*. *coli* strain K-12 as a query. The retrieved Blastp results (*e* < 10^−3^) showed the absence of YafO and HigB homologs, a limited distribution of RelE (3 hits) and a wide dissemination of YafQ and YoeB homologs. In particular, when YafQ amino acid sequence was employed as a query, 132 sequences annotated as “RelE-StbE family addiction module” or “type II toxin-antitoxin system YafQ family toxin” were identified. Similarly, when YoeB amino acid sequence was used as a query, 153 homologs annotated as “TxE-YoeB family addiction toxin” were identified. On the basis of these results, an additional PSI-BLAST search against *Lactobacillus* species commonly used in technological processes (*L*. *casei*, *L*. *paracasei* and *L*. *rhamnosus*), was carried out by using *E*. *coli* K-12 YafQ and YoeB amino acid sequences as queries. For each analysed species, we identified several putative YafQ that shared a sequence identity lower than 40%. In particular, 7 different putative YafQ toxins were identified in *L*. *casei* (YafQ_ca1-7), 6 different putative toxins in *L*. *paracasei* (YafQ_pa1-6) and 6 different putative toxins in *L*. *rhamnosus* (YafQ_rh1-6) (Fig. 1a). Some of these sequences have been identified in more than one species, with a sequence identities ranging from 73 to 100% (Supplementary Fig. S1a). The sequence identified as YafQ_rh6 is characterized by two different variants: a 94aa long full-length protein and a 61aa long N-terminal truncated form (YafQ_rh6-T). Similar PSI-BLAST analyses carried out for YoeB led to the identification of 3 sequences in *L*. *casei* (YoeB_ca1-3), 2 sequences in *L*. *paracasei* (YoeB_pa1 and 2) and 2 sequences in *L*. *rhamnosus* (YoeB_rh1 and 2) (Fig. 1b). Among these sequences, the highest sequence identity was observed between YoeB_pa1 and YoeB_rh1 (98%) and between YoeB_pa2 and YoeB_rh2 (72%) (Supplementary Fig. S1b).

To evaluate the conservation of residues crucial for enzymatic activity, YafQ and YoeB sequences from *Lactobacillus* were compared with the orthologous proteins from *E*. *coli* K-12. As shown in Fig. 1a,b, the catalytic residues are generally highly conserved and, in particular, residues with the proposed role of general acid (H87 for YafQ, H83 for YoeB) or general base (H50, H65 for YafQ and E46 for YoeB) in *E*. *coli*^11,14^ are conserved in more than 80% of the aligned sequences. For each YafQ and YoeB putative toxin, the counterpart antitoxin was identified by searching for an ORF sequence upstream of the toxin gene and the complete nucleotide sequences of the TA modules were redefined accordingly. In the case of YafQ_ca1, YafQ_ca2, YafQ_pa1 and YafQ_rh1, which share a sequence identity ≥70% (Supplementary Fig. S1a), the corresponding antitoxin was not found (orphan toxins).

To evaluate the distribution in *L*. *casei*, *L*. *paracasei* and *L*. *rhamnosus* species of TA modules *dinJ-yafQ* and *yefM-yoeB* and orphan toxins here defined, we used the BRIG software^16^ to perform a Blastn search against all the strains with a complete genome sequence in the NCBI database (Supplementary Table S1). The results show that *dinJ-yafQ* and *yefM-yoeB* systems are very rare in plasmid DNA whereas they show a wide distribution on chromosomal DNA. In particular, the *dinJ-yafQ* system was only found in the plasmid DNA of *L*. *paracasei* (5 hits) and *L*. *casei* (1 hit), whereas the *yefM-yoeB* system was not found in the plasmid DNA of all the analysed strains. Conversely, as shown in Fig. 1c, the number of TA systems identified on chromosomal DNA was significantly higher: in *L*. *casei* and *L*. *rhamnosus* the *yefM-yoeB* system was identified in 33% (yefM-yoeB_ca1) and 61% (yefM-yoeB_rh2) of the analysed strains, respectively, whereas the *yefM-yoeB* system was not found in *L*. *paracasei*. In addition, a search for the *dinJ-yafQ* module performed in *L*. *paracasei* chromosomal DNA, led to the identification of dinJ-yafQ_pa3, dinJ-yafQ_pa2 and dinJ-yafQ_pa5 in 60%, 10% and 10% of the analysed strains, respectively. Similarly, in *L*. *casei* only dinJ-yafQ_ca4 was found in 33% of the searched strains, whereas in *L*. *rhamnosus* dinJ-yafQ_rh5 was identified only in one strain and dinJ-yafQ_rh6 was identified in all the analysed strains. In the latter case, two different variants of dinJ-yafQ_rh6 were identified: a full-length toxin/antitoxin dinJ-yafQ_rh_6 module in which YafQ coding sequence overlaps the 3′-end of the *dinJ* sequence by 17 bp as reported for other TA systems^17^, and a truncated toxin variant with a deletion of 57 bp from nucleotide 42 to nucleotide 99 of the YafQ coding sequence. This putative toxin sequence has also lost the original start codon and the CDS is annotated as starting from an alternative downstream ATG or TTG codon (Supplementary Fig. S2). Translation of this proposed CDS leads to a truncated YafQ toxin (YafQ_rh6-T) lacking 33aa at the N-terminal (Fig. 1a).

The present bioinformatics analysis also shows that the orphan toxins *L*. *rhamnosus* YafQ_rh1, *L*. *casei* YafQ_ca1 and YafQ_ca2 and *L*. *paracasei* YafQ_pa1 are conserved in all the analysed strains (Fig. 1c). Further investigation is required to characterize the function of these proteins which are highly conserved even without an antitoxin within the operon. Overall, these results show that DinJ-YafQ TA systems are widely diffused in *L*. *casei*, *L*. *paracasei* and *L*. *rhamnosus* species commonly used in biotechnological processes.

### PCR-based screening for *dinJ-yafQ* TA system in *Lactobacillus* strains isolated from food

**Figure 2:**
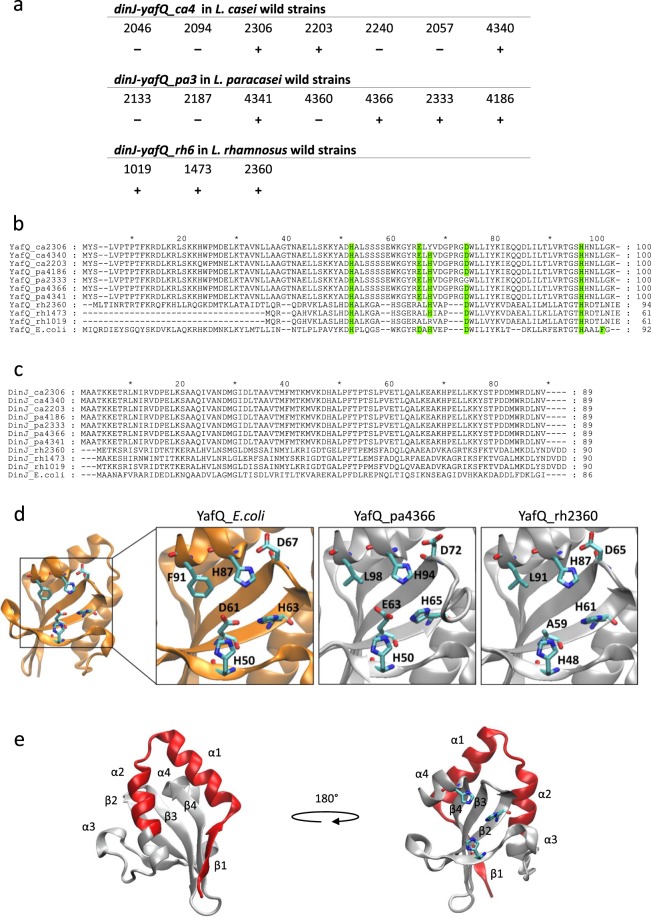
Identification and sequence alignment of *dinJ-yafQ* TA systems in wild *Lactobacillus* strains. (**a**) Outcome of PCR-based screening on wild *Lactobacillus* strains: (+) positive strains, (−) negative strains. (**b**) Sequence alignment of YafQ toxins identified in *L*. *casei*, *L*. *paracasei* and *L*. *rhamnosus* wild strains and compared with *E*. *coli* YafQ (UniProt Q47149). Catalytic residues are highlighted in green. (**c**) Sequence alignment of DinJ antitoxins identified in *L*. *casei*, *L*. *paracasei* and *L*. *rhamnosus* wild strains and compared with *E*. *coli* DinJ (UniProt Q47150). (**d**) Structure representation and close view of *E*. *coli* K-12 YafQ active sites (orange; PDB ID: 4ml2) and homology models of YafQ_pa4366 and YafQ_rh2360 (grey) with predicted catalytic residues shown in sticks. (**e**) Front and rear views of YafQ_rh2360 homology model with the N-terminal region lacking in YafQ_rh1473 highlighted in red. Catalytic histidines are shown in sticks.

To assess the presence of *dinJ-yafQ* system in *L*. *casei*, *L*. *paracasei* and *L*. *rhamnosus* species, a PCR-based screening was performed on 17 wild strains isolated from dairy products and the results are summarized in Fig. 2a. PCR primers were designed to specifically identify dinJ-yafQ_ca4, dinJ-yafQ_pa3 and dinJ-yafQ_rh6 which, based on the bioinformatics analysis, are the most frequent systems in *L*. *casei*, *L*. *paracasei* and *L*. *rhamnosus* respectively. The PCR-based screening allowed us to identify 10 complete *dinJ-yafQ* TA modules among the analysed strains. In particular, dinJ-yafQ_ca4 was found in three out of seven strains of *L*. *casei*, dinJ-yafQ_pa3 was found in four out of seven strains of *L*. *paracasei*, and dinJ-yafQ_rh6 was identified in all the three *L*. *rhamnosus* strains screened. In this latter case, the full-length variant was found in strain 2360, while the truncated variant was found in the strains 1473 and 1019.

The *dinJ-yafQ* TA modules identified in *L*. *casei*, *L*. *paracasei* and *L*. *rhamnosus* were sequenced (Supplementary Fig. S2) and the corresponding YafQ amino acid sequences were aligned with the *E*. *coli* YafQ (Fig. 2b). In all cases, sequence identity with *E*. *coli* YafQ was low, ranging from 20% to 37% as shown in Supplementary Fig. S3. The same figure also shows that sequence identity between *L*. *casei* and *L*. *paracasei* is close to 100% whereas sequence identity between *L*. *casei*/*paracasei* and *L*. *rhamnosus* is in the range 21–34%. Comparison of the *E*. *coli* YafQ catalytic residues (H50, H63, H87, D61, D67 and F91) with those found in the *Lactobacillus* strains, shows that the three histidines are highly conserved with the exception of YafQ_ca2306 and YafQ_rh1019, where H63 has been substituted with Y and R, respectively. D61 has been substituted with E in all the *L*. *casei* and *L*. *paracasei* strains and with A in *L*. *rhamnosus* strains. D67 is conserved in all strains except in YafQ_pa2333 where it has been substituted with G. This substitution has been found in 20% of the *L*. *paracasei* strains identified in the databank harbouring a dinJ-yafQ_pa3 TA system (Supplementary Fig. S4). Finally, F91 is replaced by leucine in all the analyzed *Lactobacillus* strains.

Homology modelling was employed to build the three-dimensional structure models of YafQ_pa4366 and YafQ_rh2360 using the structural coordinates of *E*. *coli* YafQ (PDB ID: 4ML2) as a template (see Materials and Methods). Figure 2d shows that the position of the catalytic residues within the active site of the three toxin proteins considered is conserved. Figure 2e depicts two orientations of the modelled YafQ_rh2360 in which the N-terminal region lacking in YafQ_rh1473 is highlighted in red. Interestingly, the deleted amino acid sequence corresponds to α-helices 1 and 2 that have been proposed to interact with the antitoxin DinJ^12^ and with the 16S rRNA^11^.

### Toxicity assays of the identified *L*. *paracasei* YafQ

Toxicity assays were conducted in *E*. *coli* by analysing bacteria growth over time both in liquid and solid media. To this end the YafQ coding sequences of *L*. *paracasei* strains 4366 and 2333 (YafQ_pa4366 and YafQ_pa2333), which differ only for the catalytic residue D72 (G72 in strain 2333), were cloned into the lactose-inducible expression vector pET11b and transformed in *E*. *coli* C41(DE3) pLysS cells (Materials and Methods).

**Figure 3:**
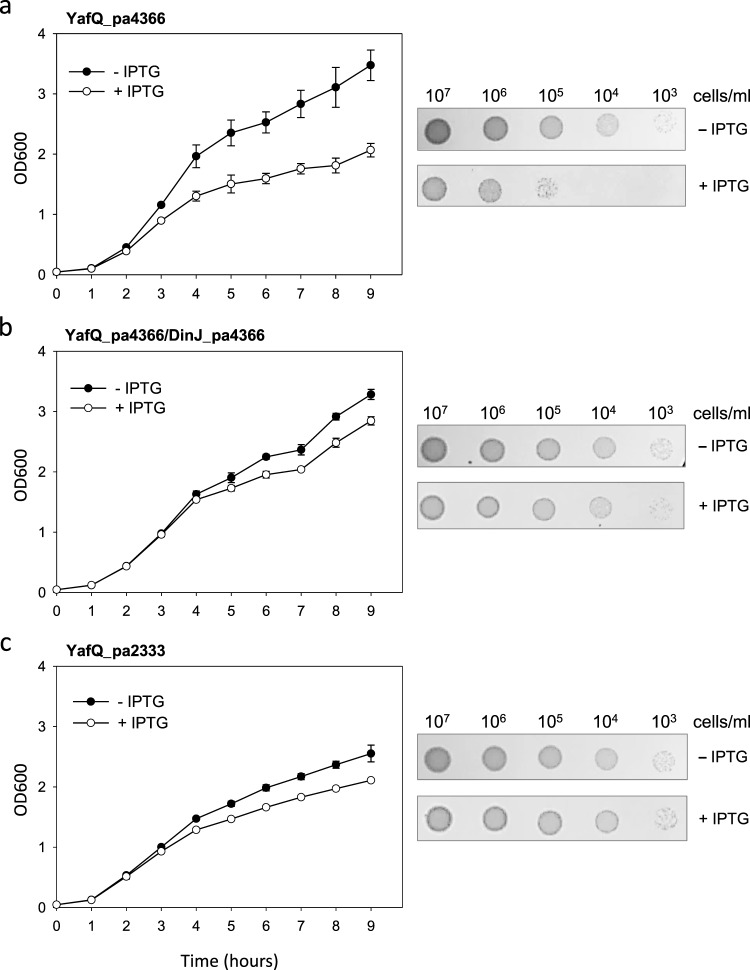
Toxicity assays of YafQ_pa4366 and YafQ_pa2333 identified in *L*. *paracasei*. Growth curves (left) and spot assay (right) of recombinant *E*. *coli* C41(DE3) pLysS strain harbouring an inducible YafQ_pa4366 gene (**a**), inducible YafQ_pa4366 and DinJ_pa4366 genes (**b**), and an inducible YafQ_pa2333 gene (**c**). In all cases, closed circles represent growth in the absence of IPTG and open circles represent growth in the presence of IPTG. Each data point represents the mean value ± SEM of three independent experiments. See Methods for experimental details.

As shown in Fig. 3a, *E*. *coli* transformed with the vector containing the YafQ_pa4366 coding sequence show a cell growth significantly inhibited upon induction with IPTG. This result demonstrates that the predicted *L*. *paracasei* YafQ protein has a strong inhibitory effect on the *E*. *coli* growth, both in liquid and solid media. To validate the whole TA system identified in *L*. *paracasei* strain 4366, the putative antitoxin DinJ_pa4366 coding sequence was cloned into the lactose-inducible expression vector pET28b and co-transformed in *E*. *coli* C41(DE3) pLysS cells. As shown in Fig. 3b, the co-expression of YafQ toxin and DinJ antitoxin restores regular growth almost completely, both in liquid and solid media. Interestingly, when *E*. *coli* cells were transformed with pET11b containing the YafQ_pa2333 coding sequence (carrying the D72G substitution) no growth inhibition was observed upon IPTG induction (Fig. 3c). These results demonstrate that the two ORF identified in *L*. *paracasei* 4366 behave as toxin/antitoxin proteins, validating the hypothesis that they are the components of a DinJ-YafQ TA system. This idea is further supported by the observation that a D72G mutation in the YafQ sequence of *L*. *paracasei* 2333 abolishes toxicity in agreement with mutagenesis experiments carried out on *E*. *coli* YafQ in which a D67A substitution led to the same effect^11^.

### Fluorescence microscopy analysis of *E*. *coli* cells expressing *L*. *paracasei* YafQ

To gather insights on the mechanism of action of the identified *L*. *paracasei* DinJ-YafQ TA system, fluorescence microscopy analysis was employed to investigate the behaviour of *E*. *coli* cell expressing the toxin. First, a set of experiments were performed using 4′,6-diamidino-2-phenylindole (DAPI) and ethidium bromide (EtBr) staining to assess membrane integrity upon toxin expression. This protocol combines the membrane-permeable fluorescent dye DAPI, which stains all bacteria, with the membrane-impermeable fluorescent dye EtBr, which permeates only bacteria with damaged membranes^18^. *E*. *coli* C41(DE3) pLysS cells grown for 3, 6 or 9 hours in the presence of IPTG and thus expressing the toxic protein YafQ_pa4366, when stained with DAPI/EtBr display only the blue fluorescence signal characteristic of DAPI with no evidence for the red EtBr signal (Supplementary Fig. S5a). This result suggests that growth inhibition observed in Fig. 3a is not associated with a membrane damage, further supporting the hypothesis that the identified TA module encompass a type II toxin.

**Figure 4:**
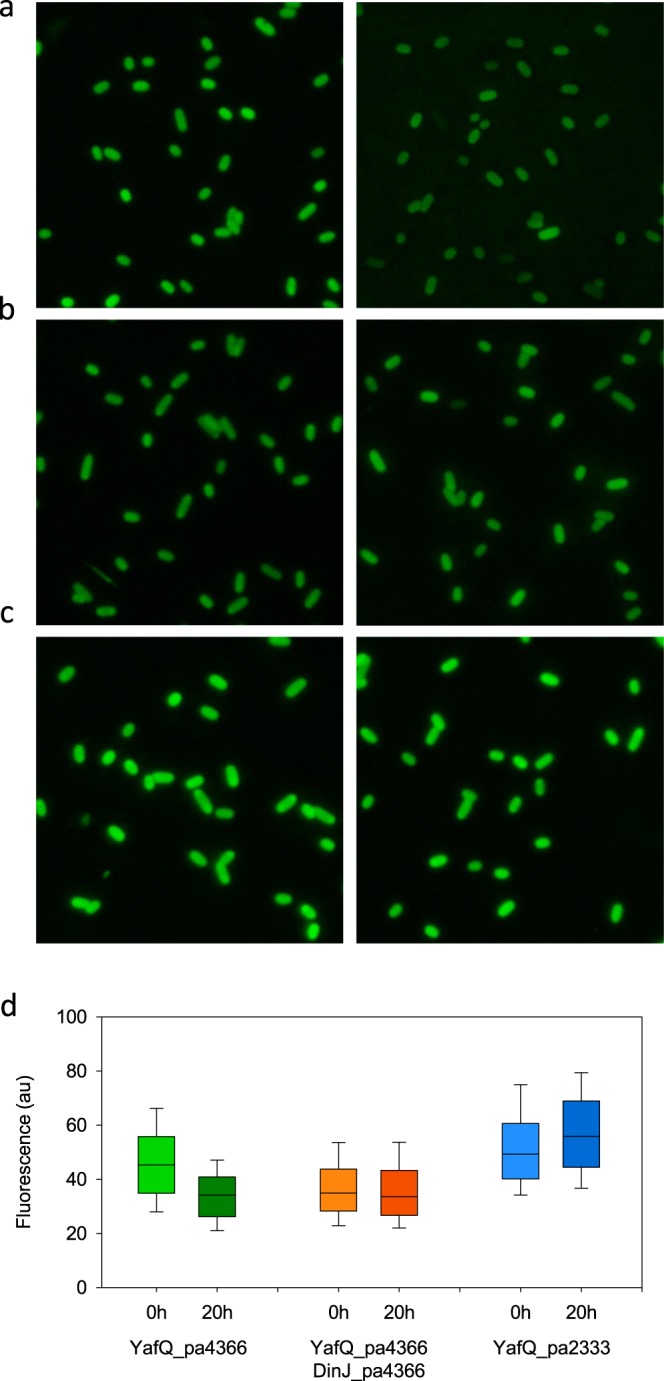
Fluorescence microscopy of *E*. *coli* C41(DE3) pLysS cells expressing *L*. *paracasei* YafQ and DinJ stained with ThT and imaged before (left) and after (right) 20 hours incubation. (**a**) Cells producing YafQ_pa4366 toxin, (**b**) cells producing YafQ_pa4366 toxin and DinJ_pa4366 antitoxin, (**c**) cells producing YafQ_pa2333 non-toxic protein. (**d**) Box-plot of the cell average fluorescence intensity relative to the six sets of images: green boxes refer to the conditions in (**a**) orange boxes refer to the conditions in (**b**) blue boxes refer to the conditions in (**c**). The boundaries of the box plot indicate the 25th and 75th percentile of the distribution, the horizontal line within the box represents the median and the error bars show the 10th and 90th percentile of the distribution. The total number of cells in each box-plot from left to right is: 1757, 1057, 1929, 1902, 1596, 1641. Each pair of box-plots was subjected to the Mann–Whitney U test to verify the statistical significance of the difference between the two distributions. Fold changes (FC) and probability (*P*) values are: FC = 0.754 *P* ≤ 0.001 for YafQ_pa4366 (green boxes); FC = 0.960 *P* = 0.011 for YafQ_pa4366/DinJ_pa4366 (orange boxes); FC = 1.134 *P* ≤ 0.001 for YafQ_pa2333 (blue boxes).

Conserved amino acids within the YafQ active site of the identified *Lactobacillus* toxins, suggest a possible RNase activity of the protein. To further investigate this aspect, we probed the RNA metabolism *in vivo* with thioflavin T (ThT) fluorescence dye which has been shown to stain preferentially bacterial total RNA rather than genomic DNA at concentrations that are not toxic for *E*. *coli* growth^19^. Under these conditions, *E*. *coli* C41(DE3) pLysS cells expressing the YafQ_pa4366 toxin show a visible decrease of fluorescence after the 20-hour incubation time (Fig. 4a). A cumulative fluorescence analysis performed over several thousand cells shows that the distribution of the cell average intensity shifts to lower values after the 20-hour incubation (Fig. 4d green boxes). Conversely, *E*. *coli* C41(DE3) pLysS cells co-expressing the YafQ_pa4366 toxin and the DinJ_pa4366 antitoxin show no change in fluorescence over an incubation time of 20 hours (Fig. 4b,d orange boxes). A similar result was obtained with *E*. *coli* C41(DE3) pLysS cells expressing the non toxic YafQ_pa2333 protein (Fig. 4c,d blue boxes). Overall, these results indicate that the identified *Lactobacillus* YafQ is capable to interfere with the RNA metabolism strengthening the hypothesis of a possible RNase activity. The results also corroborate the activity of *L*. *paracasei* 4366 TA system and highlight the importance of the mutated catalytic residue.

### Toxicity assays of the identified *L*. *rhamnosus* YafQ

**Figure 5:**
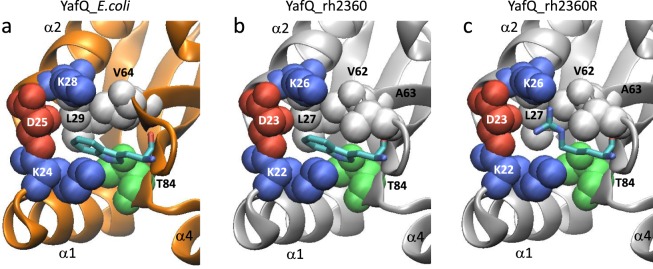
Close view of the amphipathic pocket surrounding the tryptophan undergoing mutation to arginine in YafQ_rh2360. (**a**) *E*. *coli* YafQ (PDB ID: 4ml2). (**b**) Homology model of YafQ_rh2360. (**c**) Homology model of YafQ_rh2360R. In all close views the tryptophan and the arginine are shown in sticks, hydrophobic residues are shown in light grey space-fill while charged and polar residues are in colour.

As shown in Fig. 2, we identified three strains of *L*. *rhamnosus* harbouring the DinJ-YafQ TA module: one encompassing the full-length toxin (YafQ_rh2360) and two containing a truncated version of YafQ (YafQ_rh1473 and YafQ_rh1019). Among these, YafQ_rh2360 and YafQ_rh1473 represent the full-length and truncated version of the toxin sharing the same putative catalytic residues and, therefore, they have been chosen for further characterization. To this end, the corresponding coding sequences were cloned into the lactose-inducible expression vector pET11b (see Methods). Cloning of the truncated YafQ_rh1473 was immediately successful as confirmed by DNA sequencing. Conversely, cloning of the wild-type full-length YafQ_rh2360 could not be achieved because of the many different mutations always found in the recombinant plasmids, despite of the many attempts and protocols applied. The most frequent point mutation observed was a substitution of the conserved W66 with an arginine. Several attempts to retro-mutate the W66R mutant were unsuccessful as well. Structural analysis of *E*. *coli* YafQ shows that this tryptophan lies in an amphipathic pocket behind the active site (Fig. 5a). A structural homology model of YafQ_rh2360R predicts R66 in a similar amphipathic pocket pointing towards D23 (Fig. 5b). Thus, given the impossibility of cloning the wild-type YafQ_rh2360 coding sequence and because the W66R mutation does not seem to be have a dramatic effect on protein stability, we decided to characterize the W66R mutant (YafQ_rh2360R).

**Figure 6:**
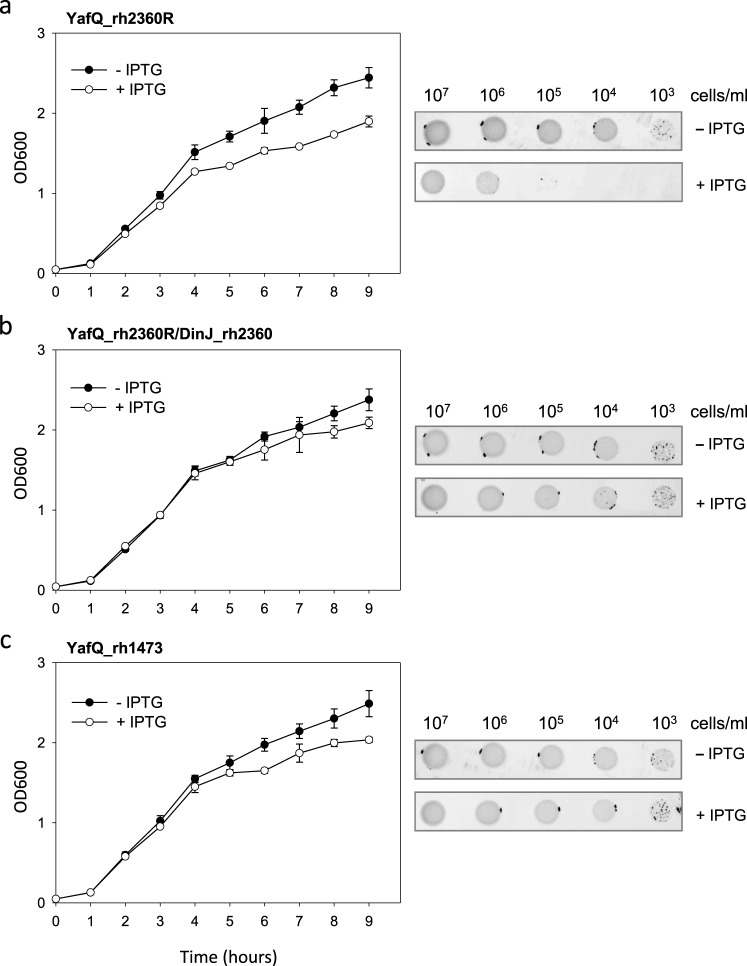
Toxicity assays of YafQ_rh2360R and YafQ_rh1473 identified in *L*. *rhamnosus*. Growth curves (left) and spot assay (right) of recombinant *E*. *coli* C41(DE3) pLysS strain harbouring an inducible YafQ_rh2360R gene (**a**), inducible YafQ_rh2360R and DinJ_rh2360 genes (**b**), and an inducible YafQ_rh1473 gene (**c**). In all cases, closed circles represent growth in the absence of IPTG and open circles represent growth in the presence of IPTG. Each data point represents the mean value ± SEM of three independent experiments. See Methods for experimental details.

As shown in Fig. 6a, when *E*. *coli* was transformed with the pET11b vector containing the YafQ_rh2360R coding sequence, cell growth in liquid medium was inhibited upon IPTG induction, although the inhibition effect was less compared to YafQ_pa4366. The lower toxicity of YafQ_rh2360R could be due to the W66R substitution introduced during cloning, however, in solid medium the inhibitory effect of YafQ_rh2360R is comparable to that observed for YafQ_pa4366. To evaluate the ability of the putative antitoxin DinJ_rh2360 to contrast YafQ_rh2360R activity, we monitored the growth of double transformed *E*. *coli* C41(DE3) pLysS cells upon IPTG induction. The results show that the co-expression of DinJ_rh2360 is sufficient to cancel the inhibitory effect of YafQ_rh2360R and to restore a normal growth both in liquid and in solid media (Fig. 6b). Thus, the activity of this *L*. *rhamnosus* 2360 TA module is in accordance with that of *L*. *paracasei* 4366 described above (Fig. 3a,b).

Similar growth assays were performed with *E*. *coli* C41(DE3) pLysS cells expressing the truncated form of the toxin (YafQ_rh1473). As shown in Fig. 6c, the expression of the truncated YafQ_rh1473 displays little inhibitory effect in liquid medium and no inhibitory effect on solid medium, albeit the predicted catalytic residues are conserved (Fig. 2b). These results are consistent with the structural model proposed for *E*. *coli* YafQ in complex with the ribosome, showing that α-helices 1 and 2 of YafQ directly contact the 16S rRNA^11^. However, the reduced or absent inhibitory effect can also be explained with loss of the native folding due to the 33aa N-terminal truncation of YafQ_rh1473 (Fig. 2e).

### Fluorescence microscopy analysis of *E*. *coli* cells expressing *L*. *rhamnosus* YafQ

A fluorescence microscopy analysis of *E*. *coli* cells expressing *L*. *rhamnosus* YafQ was performed by using DAPI and EtBr staining as described above. *E*. *coli* cells grown in the presence of IPTG, display only the blue fluorescence signal of DAPI with no evidence for the red EtBr signal (Supplementary Fig. S5b). As in the case of *L*. *paracasei* YafQ_pa4366, this observation suggests absence of membrane damage upon the expression of YafQ_rh2360 toxin.

**Figure 7:**
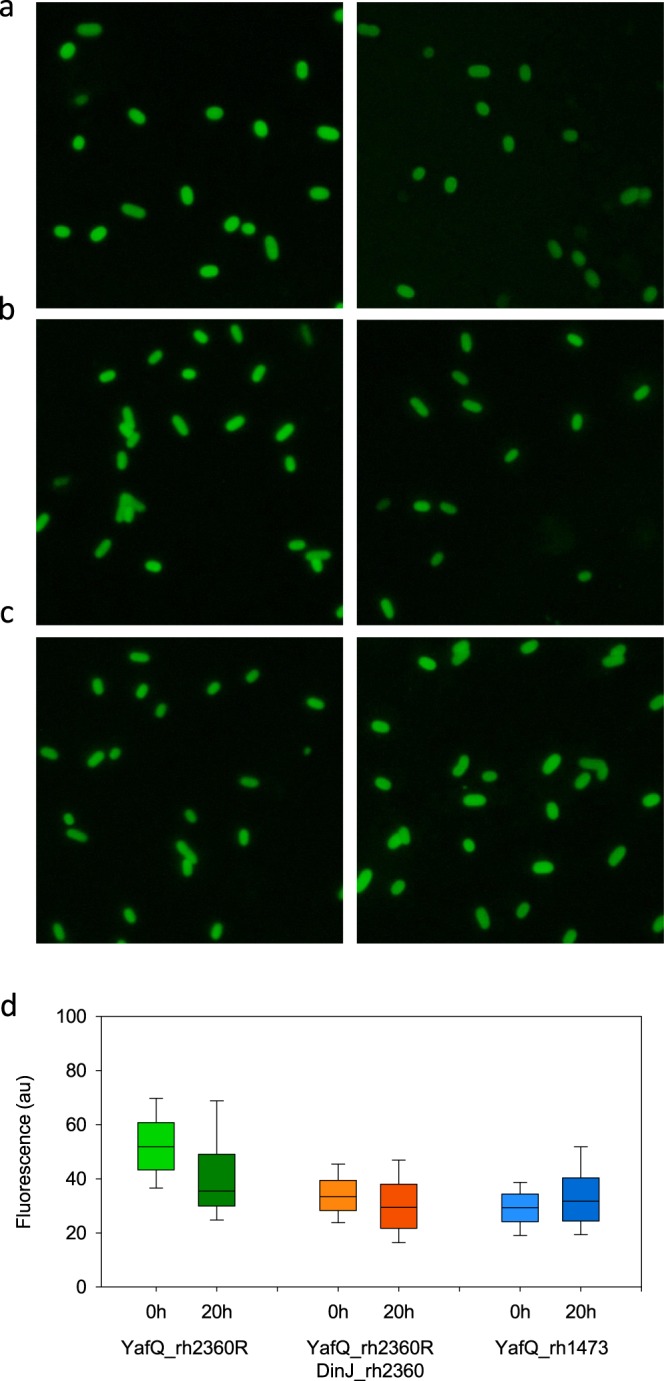
Fluorescence microscopy of *E*. *coli* C41(DE3) pLysS cells expressing *L*. *rhamnosus* YafQ and DinJ stained with ThT and imaged before (left) and after (right) 20 hours incubation. (**a**) Cells producing YafQ_rh2360R toxin, (**b**) cells producing YafQ_rh2360R toxin and DinJ_rh2360 antitoxin, (**c**) cells producing YafQ_rh1473 truncated toxin. (**d**) Box-plot of the cell average fluorescence intensity for each of the six sets of images: green boxes refer to the conditions in (**a**) orange boxes refer to the conditions in (**b**) blue boxes refer to the conditions in (**c**). The boundaries of the box plot indicate the 25th and 75th percentile of the distribution, the horizontal line within the box represents the median and the error bars show the 10th and 90th percentile of the distribution. The total number of cells in each box-plot from left to right is: 1746, 551, 1478, 773, 1338, 1538. Each pair of box-plots was subjected to the Mann–Whitney U test to verify the statistical significance of the difference between the two distributions. Fold changes (FC) and probability (*P*) values are: FC = 0.684 *P* ≤ 0.001 for YafQ_rh2360R (green boxes); FC = 0.884 *P* ≤ 0.001 for YafQ_rh2360R/DinJ_rh2360 (orange boxes); FC = 1.080 *P* ≤ 0.001 for YafQ_rh1473 (blue boxes).

The hypothesized RNase activity of the identified *L*. *rhamnosus* YafQ toxins was investigated *in vivo* by using ThT fluorescence probe in a set of experiments similar to those described above for *L*. *paracasei* toxins. As shown in Fig. 7a, the ThT fluorescence of *E*. *coli* cells expressing the YafQ_rh2360 decreases after the 20-hour incubation time. This observation is confirmed by the box-plot shown in Fig. 7d (green boxes) which represents the distribution of the cell average intensity. *E*. *coli* cells co-expressing the YafQ_rh2360 toxin and the DinJ_rh2360 antitoxin show a lower decrease in ThT fluorescence over the same incubation time (Fig. 7b,d orange boxes). Conversely, no change in ThT fluorescence was observed with *E*. *coli* cells expressing the YafQ_rh1473 truncated toxin (Fig. 7c,d blue boxes). These results suggest that the toxicity of the identified *L*. *rhamnosus* YafQ is likely related to its RNase activity. The results also support the antagonist activity of DinJ_rh2360 and the loss of activity of the truncated protein.

## Discussion

Toxins belonging to type II TA systems interfere with essential cellular processes, like protein synthesis, DNA replication or peptidoglycan synthesis. Under stress conditions, the activation of type II TA systems induces a dormant state in which cellular processes are stopped or significantly slowed down. While TA systems are well studied in pathogenic bacteria, little information is available for toxin-antitoxin systems in lactic acid bacteria widely used in industrial applications, such as fermentation processes, functional food production, nutraceutical and pharmaceutical preparations. LAB are also associated with the human gastrointestinal tract participating in the composition of gut microbiota. Given the important role played by LAB in biotechnological processes and in human health, the comprehension of LAB TA systems that can affect dynamics and viability of the bacterial population, is of great interest.

In this work we present a bioinformatics analyses of LAB sequences which led to the identification of a large number of type II TA systems comprising a ribosome-dependent RNA interferase, among which DinJ-YafQ seems the most widespread. By using a PCR-based screening of LAB wild strains isolated from dairy products, we identified several DinJ-YafQ modules in *L*. *casei*, *L*. *paracasei* and *L*. *rhamnosus* species. Among the identified TA systems, two from *L*. *paracasei* and two from *L*. *rhamnosus* were characterized for their toxin/antitoxin activity in *E*. *coli*. In particular, the TA systems DinJ-YafQ_pa4366 and DinJ-YafQ_pa2333 from *L*. *paracasei* were chosen because their toxins differ only for a residue at position 72: aspartate in YafQ_pa4366; glycine in YafQ_pa2333 (Fig. 2b). In *E*. *coli* YafQ, this residue corresponds to D67, an amino acid probably involved in catalysis as shown by mutagenesis and structural studies^11^.

The toxicity assays reported in this study show that the expression of YafQ_pa4366 inhibits *E*. *coli* growth both in liquid and solid medium. Toxin activity was however cancelled by the co-expression of the identified antitoxin DinJ_pa4366, thus validating the *L*. *paracasei* TA module as a whole. By using a recently developed fluorescence microscopy method^19^, based on the use of Thioflavin T as a probe to monitor RNA metabolism *in vivo*, we analysed the RNase activity of the identified YafQ toxins. Thioflavin T is widely used to detect amyloid fibril formation *in vitro*^20^ and it is also known to bind G-quadruplex DNA^21^, however, this is the first application of this fluorescence approach to asses *in vivo* RNA dynamics in bacteria expressing a type II toxin. The fluorescence microscopy analysis of *E*. *coli* cells expressing YafQ_pa4366 show that ThT fluorescence decreases after an incubation time of 20 hours, while it remains constant when the toxin is co-expressed with DinJ_pa4366 antitoxin. These results strongly suggest that the identified YafQ_pa4366 toxin can act as ribonuclease, in accordance with the activity shown for *E*. *coli* YafQ^8,11^.

Interestingly, the expression of YafQ_pa2333 toxin carrying a D72G mutation, does not affect *E*. *coli* growth either in liquid or solid medium. Likewise, ThT fluorescence remains constant or shows a slight increase after a 20-hour incubation. This observation highlights the essential role of D72 in the *L*. *paracasei* YafQ toxin activity and further supports the hypothesis that these toxins act as RNases. It also raises the question regarding the biological function of YafQ_pa2333 protein which has lost toxicity but it maintains high sequence identity with the wild type YafQ. An alignment among homologous YafQ_pa3 of *L*. *paracasei* retrieved from the NCBI database (Supplementary Fig. S4), shows that 3 out of 15 strains carry a YafQ toxin with the D72G mutation. Regardless of their toxicity all YafQ_pa3 proteins found in the NCBI database or identified in the present study, harbour a highly conserved antitoxin protein (Fig. 2C). This sequence conservation suggests a biological function other than the endoribonuclease activity of the toxin or the toxin-neutralizing action of the antitoxin. For instance, it has been shown that beside direct protein-protein interaction, DinJ alone or in complex with YafQ can repress mRNA transcription of the TA operon by binding to a promoter operator site^8,9^. In addition, it has been shown that type II toxins can also regulate transcription of other TA operons, thus exhibiting the potential to form cross-activation networks.

In this work we have also characterized two YafQ toxins from *L*. *rhamnosus*, YafQ_rh2360 and YafQ_rh1473 which represent the full-length and the N-terminal truncated polypeptide, respectively, of the dinJ-yafQ_rh6 group. Regarding *L*. *rhamnosus* YafQ from strain 2360, it should be noted that the characterized toxin (YafQ_rh2360R) has a W66R substitution because none of the attempts carried out to clone the wild type sequence were successful. In liquid medium, YafQ_rh2360R toxin was able to inhibit *E*. *coli* growth but to a less extend compared to *L*. *paracasei* YafQ_pa4366, whereas a similar toxicity is shown in solid medium. Our working hypothesis is that wild type YafQ_rh2360 is too toxic to be expressed in *E*. *coli* and that the mutated form YafQ_rh2360R has an attenuated activity. Co-expression of the antitoxin DinJ_rh2360 abolishes YafQ_rh2360R toxicity in *E*. *coli* thus indicating that as in the *L*. *paracasei* strain, *L*. *rhamnosus* DinJ-YafQ_rh2360 is a functional TA system and the ThT fluorescence analysis supports the RNase activity of the toxin.

The truncated form of YafQ identified in *L*. *rhamnosus* 1473 has very little toxicity in *E*. *coli* as indicated by growth assays and by ThT fluorescence analysis. These results suggest that the YafQ N-terminal sequence is crucial for toxicity in agreement with the structural model of the complex between the 70S ribosome and *E*. *coli* YafQ showing that the α1 and α2 helices contact the 16S rRNA^11^. Furthermore, structural studies of the *E*. *coli* DinJ-YafQ complex have demonstrated that YafQ N-terminal is involved in the interaction with DinJ^12^.

In the present study we demonstrate that the type II TA system DinJ-YafQ is widely distributed among *L*. *casei*, *L*. *paracasei* and *L*. *rhamnosus* species. Among the identified TA systems, we found strains with a functional DinJ-YafQ module and strains in which YafQ has lost its toxicity although maintaining high sequence identity with wild type YafQ and a likely functional antitoxin protein. The biological significance of this sequence conservation may be related to the ability of these proteins to act as transcription regulators with a potential to form cross-activation networks.

## Methods

### Bacterial strains and plasmids

*Lactobacillus* strains employed in this work have been isolated from dairy products (Supplementary Table S2), and belong to the bacterial strain collection of the Food and Drug Department of the University of Parma. *Escherichia coli* strains and plasmids used for cloning and toxicity assays are listed in the Supplementary Table S3. *Lactobacillus* strains were grown in Mann Rogosa Sharp (MRS) under anaerobiosis at 37 °C. *E*. *coli* strains were grown in Luria-Bertani (LB) broth at 37 °C in the presence of the appropriate antibiotics.

### *In silico* analysis

The first identification in *Lactobacillus* genus of toxin amino acid sequences belonging to *dinJ-yafQ*, *yafNO*, *relBE*, *higBA*, and *yefM*-*yoeB* systems was performed in the NCBI database by using the Blastp algorithm and toxin sequences from *E*. *coli* K-12 as queries. A more stringent search was performed in *L*. *casei*, *L*. *paracasei* and *L*. *rhamnosus* sequences of the NCBI database by using the PSI-BLAST algorithm and *E*. *coli* K-12 YafQ or YoeB as queries.

Nucleotide or amino acid multiple sequence alignments were performed by using MEGA7 software^22^ and ClustalW algorithm^23^. GeneDoc^24^ was used for multiple sequences alignment representations. Analysis of the abundance of dinJ-yafQ and yefM-yoeB systems in complete sequenced genomes of *L*. *casei*, *L*. *paracasei* and *L*. *rhamnosus* was performed by using Blast Ring Image Generator (BRIG) software^16^.

*L*. *paracasei* and *L*. *rhamnosus* YafQ toxins were modelled with SWISS-MODEL Server (https://swissmodel.expasy.org/)^25,26^ using the *E*. *coli* YafQ structure (PDB 4ML2) as template for modelling. Structure analyses were made by using Visual Molecular Dynamics software^27^.

### PCR-based screening

PCR primers used to amplify *L*. *casei* (dinJ-yafQ_ca4), *L*. *paracasei* (dinJ-yafQ_pa3) and *L*. *rhamnosus* (dinJ-yafQ_rh6) complete TA modules are reported in Supplementary Table S4. PCR reactions were carried out under standard conditions by using *Lactobacillus* total DNA as template and GoTaq DNA polymerase (Promega). The amplification products were verified by DNA sequencing.

### Gene cloning and *E*. *coli* transformation

The identified ORF of YafQ and DinJ proteins were amplified by PCR using total DNA extracted from *Lactobacillus* strains as template, GoTaq DNA polymerase and primers reported in Supplementary Table S4. All the amplified fragments were first cloned in pGEM-T easy vector (Promega), and subsequently cloned in the NdeI/BamHI restriction sites of the inducible expression vectors pET11b or pET28b for YafQ or DinJ coding sequences, respectively. Recombinant vectors pET11b-yafQ and pET28b-dinJ were used to transform *E*. *coli* C41(DE3) pLysS (Lucigen) by electroporation.

### Growth assays in *E*. *coli*

Activity of YafQ proteins was evaluated in *E*. *coli* C41(DE3) pLysS cells transformed with pET11b-yafQ vectors. The inhibitory activity of DinJ proteins was evaluated in the same *E*. *coli* strain co-transformed with pET11b-yafQ and pET28b-dinJ vectors. Overnight LB cultures were diluted to reach a final OD600 of about 0.05 and further grown at 37 °C for 9 hours in continuous shacking in the presence or in the absence of IPTG 1 mM. At time intervals of one hour, the OD at 600 nm was measured with the spectrophotometer. To evaluate YafQ and DinJ activity on solid media, overnight LB cultures were diluted to 1 × 10^7^, 1 × 10^6^, 1 × 10^5^, 1 × 10^4^ and 1 × 10^3^ cells/ml and 5 µl from each diluted culture were plated on LB agar in the presence or in the absence of IPTG 1 mM. Plates were incubated for 16 hours at 37 °C and growth inhibition was visually evaluated. Pictures were taken with the ChemiDoc imaging system (Bio-Rad).

### Fluorescence microscopy

*E*. *coli* liquid culture samples used for fluorescence microscopy analysis were prepared as follows: at any specific time-point of the growth, a volume of cell culture was taken such that the total amount of cells was constant and corresponded to an OD600 of 1 if resuspended in 1 ml. The harvested cells were washed 3 times with 1 ml of PBS and re-suspended in 50 μl of PBS for staining. DAPI (Sigma) and Ethidium bromide (Promega) dyes were added to a final concentration of 10 μg/mL each, followed by 5-minute incubation at RT, washed twice with 1 ml of PBS to remove the excess of dye, resuspended in 50 μl of PBS and imaged immediately. Thioflavin T (Sigma) dye was added to a final concentration of 25 μM, followed by 5-minute incubation at RT, washed twice with 1 ml of PBS to remove the excess of dye, resuspended in 50 μl of PBS and imaged immediately or after 20 hours. A reduction in the intensity of the cell fluorescence during the 20-hour incubation time is considered as a direct indication of a decreased RNA content^19^.

The glass coverslips were functionalized with poly-L ornithine (0,01%, Sigma) to favour cell adhesion. 25 μl of the PBS cell suspension were deposited onto the coverslip for 30 seconds, washed with Milli-Q water (Millipore) and dried by evaporation at RT. Fluorescence images were taken with a Nikon Eclipse E600 microscope equipped with a 100X oil immersion objective and with a Nikon DS-Fi2 digital camera. For DAPI/EtBr staining the UV-2A filter was used. For ThT staining the B2A filter was used. Due to ThT fast bleaching, cells were focused in phase contrast and images were recorded immediately after shutter opening with an exposure time of 800 msec.

For DAPI/EtBr staining, raw images were processed with ImageJ for background subtraction with a 20 pixel rolling ball radius. For ThT staining, raw images were analyzed with *ad-hoc* written Matlab scripts. Only the green channel of the RGB image was used for image processing. First, a global threshold to convert the green channel into a binary image was computed using Otsu’s method of the *graythresh* Matlab function. To increase the quality of the data set, only images with a global threshold value within one standard deviation from the mean of the dataset global threshold distribution, were analyzed. Secondly, images were binarized with the *imbinarize* Matlab function using the computed global threshold. Objects with less than 100 pixels or touching the image edge were removed. For each object, the skeletonized perimeter was determined and the filled object was used as a mask on the green channel to compute area, perimeter and mean intensity of each object. To maximize single cells detection, a morphological filter was applied: only objects with an area in the range 300–1000 pixels and with a perimeter smaller or equal than 165 pixels were retained. The mean fluorescence intensities of the selected cells were imported into Sigmaplot (Systat Software, Inc.) to construct box-plots using the standard method.

## Supplementary information

Supplementary Tables
