# Supplementary material for: Identification and first characterization of DinJ-YafQ toxin-antitoxin systems in Lactobacillus species of biotechnological interest
Source: Sci Rep. 2019 May 21;9:7645. doi: 10.1038/s41598-019-44094-6 (PMC6529426; doi:10.1038/s41598-019-44094-6)
Supplement: Supplementary file 1 — Supplementary Tables [file 41598_2019_44094_MOESM1_ESM.pdf]

Identification and first characterization of DinJ-YafQ toxin-antitoxin systems in *Lactobacillus* species of biotechnological interest

Alberto Ferrari, Stefano Maggi, Barbara Montanini, Alessia Levante, Camilla Lazzi, Yoshihiro Yamaguchi, Claudio Rivetti and Claudia Folli

Supplementary Table S1. Subset of *Lactobacillus* strains used for BRIG software analysis

Species	Strain	Chromosome Entry
L. casei	12A	NZ_CP006690.1/CP006690.1
L. casei	ATCC 393	NZ_AP012544.1/AP012544.1
L. casei	BL23	NC_010999.1/FM177140.1
L. casei	CECT 9104	NZ_LS991421.1/LS991421.1
L. casei	LC5	NZ_CP017065.1/CP017065.1
L. casei	W56	NC_018641.1/HE970764.1
L. paracasei	8700:2	NC_022112.1/CP002391.1
L. paracasei	ATCC 334	NC_008526.1/CP000423.1
L. paracasei	BD-II	NC_017474.1/CP002618.1
L. paracasei	CAUH35	NZ_CP012187.1/CP012187.1
L. paracasei	EG9	NZ_CP029546.1/CP029546.1
L. paracasei	FAM18149	NZ_CP017261.1/CP017261.1
L. paracasei	HD1.7	NZ_CP025582.1/CP025582.1
L. paracasei	HDS-01	NZ_CP026097.1/CP026097.1
L. paracasei	IIA	NZ_CP014985.1/CP014985.1
L. paracasei	JCM 8130	NZ_AP012541.1/AP012541.1
L. paracasei	KL1	NZ_CP013921.1/CP013921.1
L. paracasei	L9	NZ_CP012148.1/CP012148.1
L. paracasei	LC2W	NC_017473.1/CP002616.1
L. paracasei	LC355	NZ_CP029536.1/CP029536.1
L. paracasei	LOCK919	NC_021721.1/CP005486.1
L. paracasei	Lpc10	NZ_CP029686.1/CP029686.1
L. paracasei	N1115	NZ_CP007122.1/CP007122.1
L. paracasei	TK1501	NZ_CP017716.1/CP017716.1
L. paracasei	TMW 1.1434	NZ_CP016355.1/CP016355.1
L. paracasei	ZFM54	CP032637.1
L. paracasei	Zhang	NC_014334.2/CP001084.2
L. rhamnosus	4B15	NZ_CP021426.1/CP021426.1
L. rhamnosus	ATCC 11443	NZ_CP022109.1/CP022109.1
L. rhamnosus	ATCC 53103	NC_017482.1/AP011548.1
L. rhamnosus	ATCC 8530	NC_017491.1/CP003094.1
L. rhamnosus	BFE5264	NZ_CP014201.1/CP014201.1
L. rhamnosus	BPL5	NZ_LT220504.1/LT220504.1
L. rhamnosus	DSM 14870	NZ_CP006804.1/CP006804.1
L. rhamnosus	GG	NZ_CP031290.1/CP031290.1
L. rhamnosus	GG (ATCC 53103)	NC_013198.1/FM179322.1
L. rhamnosus	Lc 705	NC_013199.1/FM179323.1
L. rhamnosus	LOCK900	NC_021723.1/CP005484.1
L. rhamnosus	LOCK908	NC_021725.1/CP005485.1
L. rhamnosus	LR5	NZ_CP017063.1/CP017063.1
L. rhamnosus	LRB	CP016823.1
L. rhamnosus	Pen	NZ_CP020464.1/CP020464.1
L. rhamnosus	SCT-10-10-60	NZ_CP019305.1/CP019305.1

Supplementary Table S2. *Lactobacillus* strains used in this work

Lactobacillus species	**Strain**	**Source**
L. rhamnosus	1019, 1473, 2360	Parmigiano Reggiano cheese
L. casei	2046, 2094, 2057	Grana Padano cheese
L. casei	2306, 2240	Parmigiano Reggiano cheese
L. casei	2203, 4340	Milk
L. paracasei	2133, 2187	Grana Padano cheese
L. paracasei	4341, 4360, 4366, 4186	Milk
L. paracasei	2333	Parmigiano Reggiano cheese

Supplementary Table S3. *E. coli* strains and plasmids used in this work

***E. coli* strain**	**Description**	**Source**
XL1-Blue	Cloning and plasmid purification, Tet^R	Stratagene
C41(DE3) pLysS	Toxic activity assay, Cam^R	Lucigen
**Plasmid**	**Description**	**Source**
pGEM-T easy	Cloning vector	Promega
pET11b	Expression vector, IPTG inducible, Amp^R	Novagen
pET28b	Expression vector, IPTG inducible, Kan^R	Novagen
pET11b-yafQ_pa4366	YafQ CDS from *L. paracasei* 4366 cloned in pET11b	This work
pET11b-yafQ_pa2333	YafQ CDS from *L. paracasei* 2333 cloned in pET11b	This work
pET28-dinJ_ pa4366	DinJ CDS from *L. paracasei* 4366 cloned in pET28b	This work
pET11b-yafQ_rh2360	YafQ CDS from *L .rhamnosus* 2360 cloned in pET11b	This work
pET11b-yafQ_rh1473	YafQ CDS from *L .rhamnosus* 1473 cloned in pET11b	This work
pET28-dinJ_rh2360	DinJ CDS from *L. rhamnosus* 2360 cloned in pET28b	This work

Supplementary Table S4. Oligonucleotides used in this work

Primer	Sequence	Target strains
***dinJ-yafQ* identification**		
dinj-yafQ_ca4_pa3 plus	AAGTGCTGCTCAAATCGTAGC	*L. casei, L. paracasei*
dinj-yafQ_ca4_pa3 minus	AAGGTTATGATGAGATCCGGTTC	*L. casei, L. paracasei*
dinj-yafQ_rh6 plus	CCATGTTCTTAATAGCATGGG	*L. rhamnosus*
dinj-yafQ_rh6 minus	TTACTCAATGTTCAATGTATCGCG	*L. rhamnosus*
**YafQ and DinJ cloning**		
yafQ_pa plus	CATATGTATAGTCTGGTTCCGACG	*L. paracasei* 4366, 2333
YafQ_pa minus	GGATCCTATTTACCCAGAAGGTTATGA	*L. paracasei* 4366, 2333
dinJ_pa4366 plus	CATATGGCAGCCACAAAGAAAGAA	*L. paracasei* 4366
dinJ_pa4366 minus	GGATCCCTATACATTCAAGTCTCTCCAC	*L. paracasei* 4366
yafQ_rh2360 plus	CATATGTTGACGATTAATCGCACG	*L. rhamnosus* 2360
yafQ_rh1473 plus	CATATGCAGCGTCAAGGTCATGTA	*L. rhamnosus* 1473
yafQ_rh minus	GGATCCTTACTCAATGTTCAATGTATCGC	*L. rhamnosus* 2360, 1473
dinJ_rh2360 minus	GGATCCTTAATCGTCAACATCATTGTATAA	*L. rhamnosus* 2360
dinJ_rh2360 plus	CATATGGAAACAAAATCCCGTATCA	*L. rhamnosus* 2360

	YafQ_rh2	YafQ_rh3	YafQ_rh4	YafQ_rh5	YafQ_rh6	YafQ_ca1	YafQ_ca2	YafQ_ca3	YafQ_ca4	YafQ_ca5	YafQ_ca6	YafQ_ca7	YafQ_pa1	YafQ_pa2	YafQ_pa3	YafQ_pa4	YafQ_pa5	YafQ_pa6	YafQ_E.coli
YafQ_rh1	38%	21%	17%	24%	25%	74%	77%	38%	21%	17%	21%	14%	74%	38%	22%	17%	14%	21%	18%
YafQ_rh2		22%	19%	23%	29%	40%	35%	98%	26%	15%	22%	17%	40%	98%	26%	15%	17%	22%	26%
YafQ_rh3			15%	24%	19%	20%	19%	22%	25%	17%	100%	25%	20%	22%	25%	18%	25%	100%	20%
YafQ_rh4				23%	21%	18%	17%	19%	43%	20%	15%	11%	18%	19%	44%	20%	11%	15%	23%
YafQ_rh5					27%	21%	22%	23%	34%	33%	24%	24%	21%	23%	34%	33%	24%	24%	25%
YafQ_rh6						24%	25%	29%	33%	30%	19%	17%	24%	28%	33%	30%	17%	19%	27%
YafQ_ca1							74%	40%	25%	18%	20%	11%	97%	40%	25%	18%	11%	20%	19%
YafQ_ca2								35%	22%	21%	19%	14%	73%	35%	23%	21%	14%	19%	20%
YafQ_ca3									26%	15%	22%	17%	40%	97%	26%	15%	17%	22%	26%
YafQ_ca4										33%	25%	22%	25%	26%	99%	33%	22%	25%	36%
YafQ_ca5											17%	17%	18%	15%	33%	98%	17%	17%	23%
YafQ_ca6												25%	20%	22%	25%	18%	25%	100%	20%
YafQ_ca7													11%	17%	22%	17%	100%	25%	16%
YafQ_pa1														40%	25%	18%	11%	20%	19%
YafQ_pa2															26%	15%	17%	22%	26%
YafQ_pa3																33%	22%	25%	36%
YafQ_pa4																	17%	18%	23%
YafQ_pa5																		25%	16%
YafQ_pa6																			20%

	YoeB_Rh_2	YoeB_Ca_1	YoeB_Ca_2	YoeB_Ca_3	YoeB_Pa_1	YoeB_Pa_2	YoeB_E.coli
YoeB_Rh_1	61%	67%	22%	19%	98%	61%	57%
YoeB_Rh_2		60%	17%	19%	62%	72%	47%
YoeB_Ca_1			21%	21%	68%	61%	55%
YoeB_Ca_2				68%	23%	20%	24%
YoeB_Ca_3					20%	21%	19%
YoeB_Pa_1						62%	58%
YoeB_Pa_2							53%

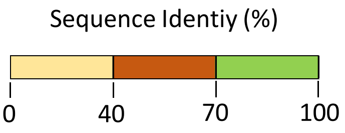

Supplementary Figure S1. Sequence identities of YafQ and YoeB toxins identified in *Lactobacillus* genus. a) Percentages of identity among YafQ aminoacid sequences retrived from PSI-BLAST and compared with *E. coli* YafQ (UniProt Q47149). b) Percentages of identity among YoeB aminoacid sequences retrived from PSI-BLAST and compared with *E. coli* YoeB (UniProt P69348).

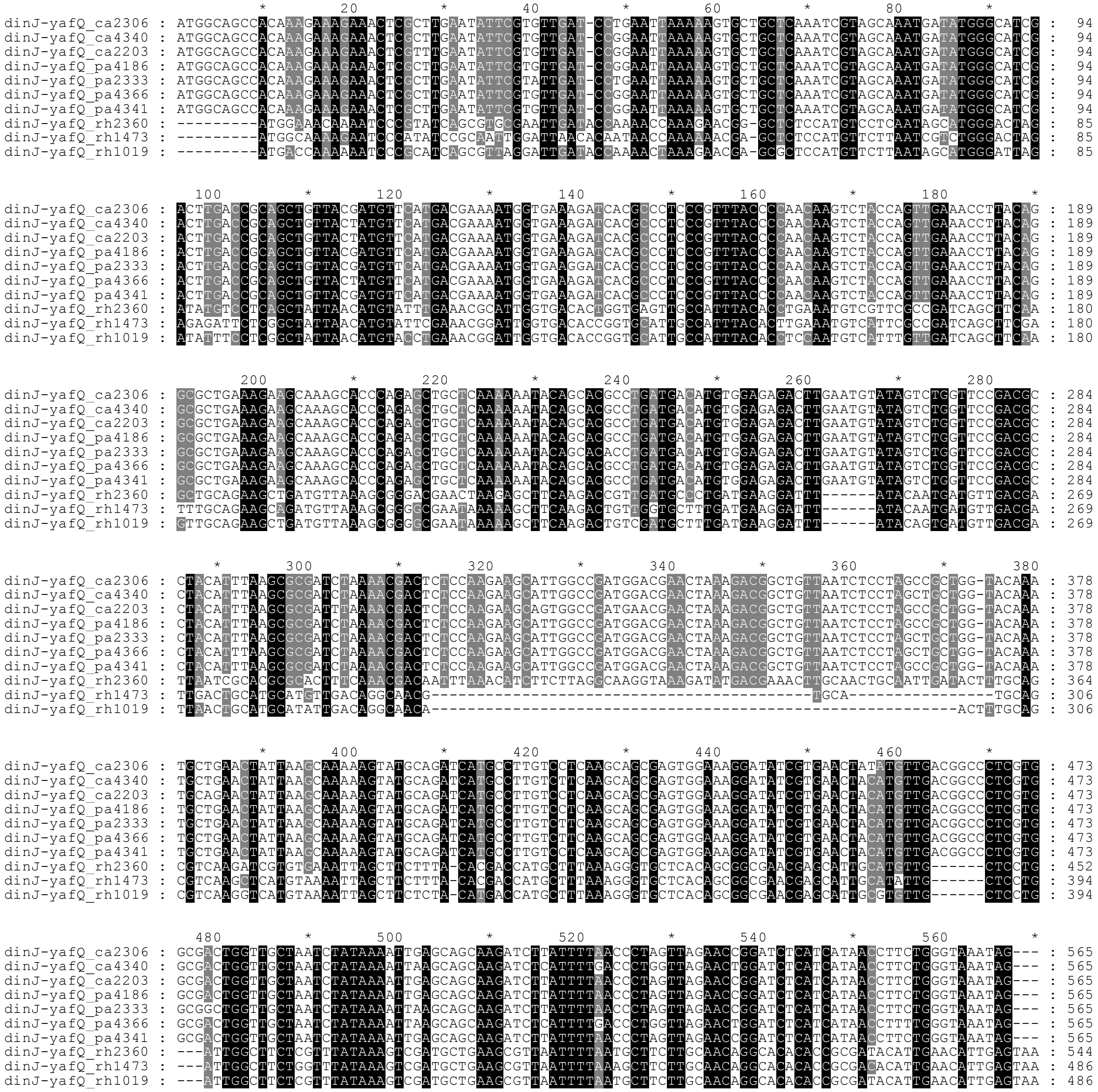

Operon	GenBank ID
dinJ-yafQ_ca2306	MK544939
dinJ-yafQ_ca4340	MK544940
dinJ-yafQ_ca2203	MK544941
dinJ-yafQ_pa4186	MK544942
dinJ-yafQ_pa2333	MK544943
dinJ-yafQ_pa4366	MK544944
dinJ-yafQ_pa4341	MK544945
dinJ-yafQ_rh2360	MK544946
dinJ-yafQ_rh1473	MK544947
dinJ-yafQ_rh1019	MK544948

Supplementary Figure S2. Nucleotide sequence alignment and GenBank ID of the identified *dinJ-yafQ* operons shown in Figure 2a.

	YafQ_ca4340	YafQ_ca2203	YafQ_pa4186	YafQ_pa2333	YafQ_pa4366	YafQ_pa4341	YafQ_rh2360	YafQ_rh1473	YafQ_rh1019	YafQ_E.coli
**YafQ_ca2306**	98%	97%	99%	97%	98%	99%	33%	21%	22%	36%
**YafQ_ca4340**		97%	99%	99%	100%	99%	34%	22%	22%	37%
**YafQ_ca2203**			98%	96%	97%	98%	34%	22%	22%	37%
**YafQ_pa4186**				98%	99%	100%	34%	22%	22%	37%
**YafQ_pa2333**					99%	98%	33%	21%	21%	36%
**YafQ_pa4366**						99%	34%	22%	22%	37%
**YafQ_pa4341**							34%	22%	22%	37%
**YafQ_rh2360**								59%	60%	29%
**YafQ_rh1473**									95%	20%
**YafQ_rh1019**										20%

	DinJ_ca4340	DinJ_ca2203	DinJ_pa4186	DinJ_pa2333	DinJ_pa4366	DinJ_pa4341	DinJ_rh2360	DinJ_rh1473	DinJ_rh1019	DinJ_E.coli
**DinJ_ca2306**	100%	100%	100%	98%	100%	100%	26%	20%	29%	24%
**DinJ_ca4340**		100%	100%	98%	100%	100%	26%	20%	29%	24%
**DinJ_ca2203**			100%	98%	100%	100%	26%	20%	29%	24%
**DinJ_pa4186**				98%	100%	100%	26%	20%	29%	24%
**DinJ_pa2333**					98%	98%	27%	21%	30%	25%
**DinJ_pa4366**						100%	26%	20%	29%	24%
**DinJ_pa4341**							26%	20%	29%	24%
**DinJ_rh2360**								75%	90%	23%
**DinJ_rh1473**									75%	20%
**DinJ_rh1019**										25%

Sequence Identiy (%)

0 40 70 100

Supplementary Figure S3. Sequence identities of YafQ and DinJ proteins found in *L. casei*, *L. paracasei* and *L. rhamnosus*. a) Percentages of identity among the identified YafQ aminoacid sequences and with *E. coli* YafQ (UniProt Q47149). b) Percentages of identity among the identified DinJ aminoacid sequences and with *E. coli* DinJ (UniProt Q47150).

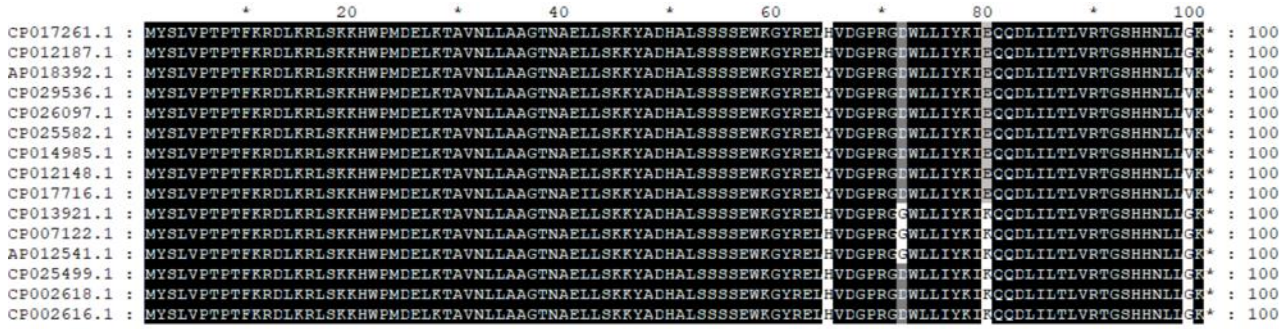

Supplementary Figure S4. Sequence alignment of YafQ_pa3 of *L. paracasei* strains retrieved from the databank.

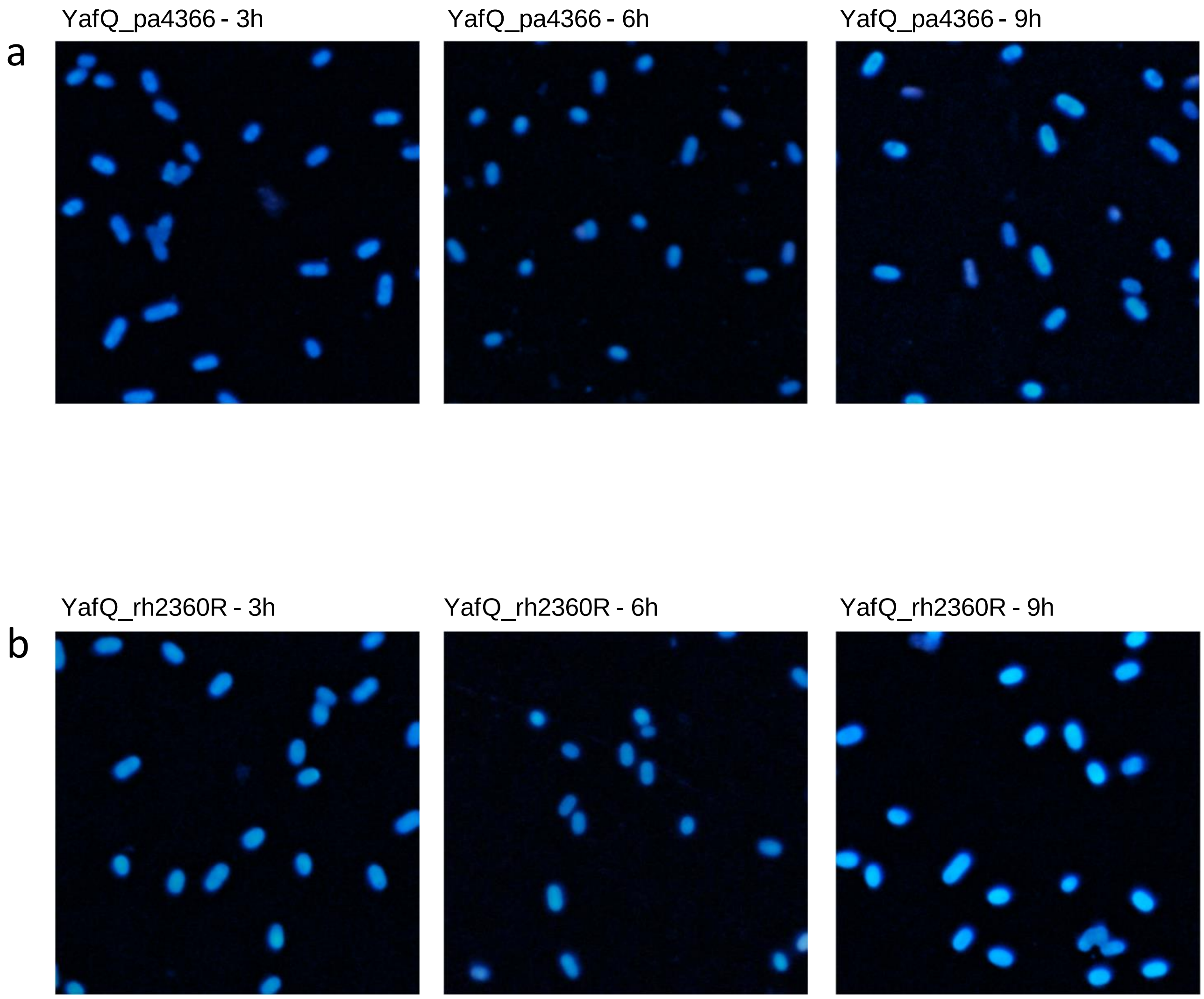

Supplementary Figure S5. Membrane integrity assay. Fluorescence microscopy of *E. coli* C41(DE3) pLysS cells expressing *Lactobacillus* YafQ and stained with DAPI/EtBr dyes. (a) Cells producing YafQ_pa4366 toxin grown in LB/IPTG medium for 3, 6 or 9 hours. (b) Cells producing YafQ_rh2360R toxin grown in LB/IPTG medium for 3, 6 or 9 hours. Absence of the red EtBr fluorescence indicates membrane integrity.
